# Their Uses and Manufacture

**Published:** 1921-06-25

**Authors:** 


### CELLULOID SPLINTS.

### Their Uses and Manufacture.

In the past few years the value of splints made of celluloid has been increasingly realised, and it has been found that they have many advantages which are not possessed by splints made of other materials. Any medical man can make a cheap and really efficient splint himself, suitable for the use of any individual patient, after a short experience of the technique. While equally valuable for most orthopædic work, these splints are perhaps especially suitable in various deformities occurring in children and in the convalescent stage of tuberculous disease of bones or joints.

The special advantages of the splints are perfect accuracy of fit; lightness (they weigh less than similar ones made of any other material); cleanliness (they can be washed); ease of application (patients can remove and reapply them without fear of putting them on wrongly, and they need no readjustment).

### Manufacture.

(a) The part of the body which the splint is to cover is encased in a thin layer composed of plaster-of-Paris bandages. This plaster case, when removed, forms a hollow mould.

(b) The mould is filled with plaster of Paris, which, when dry, forms a plaster "positive" the exact shape of the part.

(c) Layers of material are pasted all over this "positive" with coats of celluloid solution, until a celluloid case of sufficient thickness has been formed.

(d) The celluloid case is cut off the "positive," tried on to the patient, the unnecessary parts being cut away, and the splint is finished off as described below.

### (A) Taking the "Cast" or "Negative."

The part of the patient for which the splint is to be made is first smeared over with a little warm olive oil, and any long hairs are either shaved off or covered with a thin layer of gauze. If these precautions to facilitate the removal of the cast are not taken considerable pain may be produced by the plaster of Paris adhering to the skin.

The affected limb or other part of the body is then carefully held in the required position while it is encased in a thin covering of plaster-of-Paris bandages. Bandages made at home by rolling strips of book-muslin in dry plaster are preferable to those obtained ready-made. Warm water should be used in which to soak the bandages before use, so that the "setting" of the plaster may be expedited. The "cast" should not be made too thick, and should be carefully moulded around the bony points, to ensure sufficient purchase being maintained to effect fixation of the part.

The "cast," having "set," is removed by slitting it up one side, lines at right-angles to the slit being first pencilled on it, so that the exact shape may afterwards be restored. A few turns of a plaster bandage are then made, to fix together the cut edges and to obliterate all the holes except the largest. For instance, in making a splint for the trunk the holes for the neck and arms would be filled up, leaving open only the pelvic aperture.

### (B) Making the "Positive."

The interior of the "cast" is first carefully "oiled" with olive oil or a thick solution of soft soap and water. Three parts of dry commercial plaster is made into a paste with about five parts of water, and the cast is filled with it. Care is to be taken that all the hollows are filled up. When quite dry, the outer "cast" or "negative" is removed. All rough surfaces are removed from the solid "positive," and plaster is cut away to accentuate the normal mouldings of the body, and so increase its purchase on bony points. The whole "positive" is then covered with a thin smooth layer of plaster of Paris and left to dry thoroughly.

### (C) Making the Celluloid "Cast."

Two layers of vesting are then put over the "positive." These must fit very closely. They are to line the finished splint as a smooth inner layer. The celluloid solution is then prepared. Celluloid of a low nitrification is used. Celluloid scrap or thin sheets are cut up small and dissolved in acetone until a solution rather less stiff than treacle is formed. Three ounces of calcium chloride, dissolved in two ounces of boiling water, is added slowly to about seven pints of the celluloid-acetone solution. The solution, which was before clear, becomes cloudy and white, as the calcium salt of celluloid is formed. This is non-inflammable. This mixture is painted on to the vest and thoroughly rubbed in. As each coat dries another is painted on, until several coats have been applied.

A layer of book-muslin is then applied, and fixed in position with several coats of celluloid solution. This process is repeated until the required thickness is obtained, care being taken that each coat is allowed to dry thoroughly before the next one is added. In those areas where extra strength is required additional strips of muslin are fixed. The number of layers required will, of course, vary with the size of the splint. About eighteen to twenty layers will be needed in a hip or spine splint for a child of about sixteen years of age.

### (D) Fitting On and Finishing.

The celluloid is then cut off the "positive" by making an incision through it in the most suitable position for the individual case. It is then tried on the patient and edges are trimmed and all unwanted parts cut off. Where necessary, strips of "duralumin" or similar metal are moulded to the shape of the splint and riveted on where extra support is required. The splint is perforated all over with suitable-sized holes, to allow of free ventilation. The edges are then bound with soft leather. (The type commercially known as "Basils" is very suitable.) Arrangements are then made for fastening the splint on. Either straps and buckles, or else strips of leather with eyelet holes for lacing.

